# The psychological effects of preterm birth on postnatal mothers: a scoping review

**DOI:** 10.1186/s12884-025-08504-0

**Published:** 2025-11-22

**Authors:** Ganesh Handady, Suchetha S. Rao, K. Keshava Pai, S. Elstin Anbu Raj, K. Shraddha Shetty, P. Prasanna Mithra, Santosh Rai

**Affiliations:** 1 Department of Pediatrics, Kasturba Medical College Mangalore, Manipal Academy of Higher Education, Manipal, India; 2 Department of Psychiatry, Kasturba Medical College Mangalore, Manipal Academy of Higher Education, Manipal, India; 3 Centre for Evidence-informed Decision-making, Prasanna School of Public Health, Manipal Academy of Higher Education, Manipal, India; 4 Department of Obstetrics and Gynecology, Kasturba Medical College Mangalore, Manipal Academy of Higher Education, Manipal, India; 5 Department of Community Medicine, Kasturba Medical College Mangalore, Manipal Academy of Higher Education, Manipal, India; 6 Department of Radiodiagnosis and Imaging, Kasturba Medical College Mangalore, Manipal Academy of Higher Education, Manipal, India

**Keywords:** Anxiety, Depressive symptoms, Mental health, Preterm birth, Postpartum women, Psychological effects

## Abstract

**Background:**

One of five new mothers might experience psychological effects, most of which are found in the case of preterm births. This scoping review aimed to synthesize the literature on the psychological effects of preterm birth on postpartum mothers in healthcare settings.

**Methods:**

We conducted a scoping review in accordance with the Joanna Briggs Institute methodology and the Arksey and O’Malley methodological framework for scoping review. A search was conducted in PubMed (National Center for Biotechnology Information), Embase (Elsevier), and Scopus (Elsevier). The studies were screened and extracted via a predesigned data extraction form.

**Results:**

A total of 1477 articles were identified, 35 of which were eligible for full-text screening. Ultimately, 21 articles were included in the review. Thirteen of the 21 studies measured anxiety, eleven studies measured depressive symptoms, nine studies measured stress, and five studies each measured posttraumatic stress symptoms, postpartum depressive symptoms, and distress symptoms. The percentage of anxiety levels ranged between 12.3% and 83%, and the percentage of depression levels ranged between 19.3% and 90.6%.

**Conclusions:**

The most common psychological effect was anxiety, followed by depressive symptoms and stress. Gestational age, preeclampsia, infant behavior and appearance in the neonatal intensive care unit (NICU) were common risk factors. State-Trait Anxiety Inventory for anxiety, the Center for Epidemiology Studies Depression Scale for depression, and the Perceived Stress Scale: NICU for stress were frequently used screening tools.

**Supplementary Information:**

The online version contains supplementary material available at 10.1186/s12884-025-08504-0.

## Introduction

Worldwide, approximately 13% of new mothers suffer from mental illness, mainly depression. This percentage is significantly greater in developing countries, where it is 19.8% after childbirth [1]. Giving birth may turn into a traumatic experience when the mother experiences pain, fear, loss of control, and helplessness during labor, and the postpartum period may represent a time of greater psychological vulnerability. One in five new mothers might experience postpartum depressive and anxiety symptoms on the basis of relevant stressors and social circumstances. These factors are most commonly found in the case of preterm births [2, 3].

Preterm birth is defined as “neonates delivered alive before completion of 37 weeks of gestation” [4]. Every year, over 15 million babies are born preterm. Mothers of very preterm infants reported much greater levels of anxiety, stress, and depressive symptoms than mothers with full-term infants did, with up to 40% of mothers reporting depression [5]. Unanticipated preterm pregnancies and subsequent neonatal intensive care unit (NICU) admissions can be highly traumatic for parents [6, 7]. In the NICU, stressors such as the newborn’s unstable health, tubes, monitors, wires, and noise from different monitoring devices significantly impact parental psychological well-being [7].

Barnard identified the caregiver, infant and environment as the primary elements of the mother–infant interaction system [8]. In this reciprocal system, the emotions and needs of both mothers and infants continuously influence each other. Maternal mental health issues such as depression, anxiety and stress can disrupt responsiveness, leading to suboptimal emotional outcomes for infants [8]. Additionally, maternal depressive symptoms alter children’s neurobiological responses to stress and impact areas of the brain related to executive function [9]. Parental stress, depression, anxiety, and fatigue can alter parenting styles, parent–infant interactions, perceptions of parental competence, and infant outcomes such as health, infant temperament, fine motor outcomes, cognitive development, and emotional regulation [5, 10, 11]. Social risk, a history of mental illness in mothers, interpersonal factors, neonatal factors such as disability, increased duration of incubator days, and intraventricular hemorrhage have been identified as risk factors for psychological effects following very preterm birth [12].

It is critical to assess maternal psychological responses following preterm birth, as maternal mental health is associated with children’s psychological and physical development [2]. This review aims to synthesize existing evidence regarding the psychological effects of preterm birth on postnatal mothers, risk factors and assessment tools used in health care settings.

Our objectives were as follows:

1. To determine the psychological effects of preterm birth on postnatal mothers in healthcare settings.
2. To identify the risk factors predisposing mothers to these psychological effects.
3. To identify the common assessment tools used in health care settings to evaluate these psychological effects.

## Methods

Using the scoping review methodology, we investigated the relevant literature available to better understand the psychological effects of preterm birth on mothers. The proposed scoping review was conducted in accordance with the Joanna Briggs Institute (JBI) methodology for scoping reviews [13]. We followed the six-step methodological framework for scoping review suggested by Arksey and O’Malley [14]. This scoping review was reported in accordance with the Preferred Reporting Items for Systematic Reviews and Meta-Analyses (PRISMA) Extension for Scoping Reviews Checklist [15] ( Supplementary file 1) .

### Eligibility criteria

Studies performed in healthcare settings reporting psychological effects on postnatal mothers following preterm birth were included. Studies were included if they reported at least one of the following psychological effects among postnatal mothers following preterm birth: anxiety, depression, postpartum depression, stress, posttraumatic stress or distress. Analytical and descriptive cross-sectional studies, prospective and retrospective cohort studies, case‒control studies and longitudinal studies in which the initial psychological screening was performed in healthcare settings were included.

Studies screening for psychological effects following preterm delivery outside the healthcare setting (home, community setting, etc.) and studies assessing psychological effects following preterm delivery in mothers with preexisting psychiatric illness were excluded. Qualitative studies, review articles, texts, opinion papers, case reports, randomized control trials, interventional studies, conference proceedings, and gray literature were excluded.

### Database selection and search strategy

PubMed, Scopus, and Embase were chosen because of their broad subject indexing and comprehensive coverage of biomedical and psychological literature. An extensive search strategy was developed by identifying the relevant keywords, including “preterm birth, premature birth, psychological effects, mothers”, which were combined with appropriate MeSH terms from the PubMed database. The search terms were combined via appropriate Boolean operators such as “AND” and “OR”. The search terms used in PubMed were “((“premature birth“[MeSH Terms] OR preterm birth[Text Word]) AND (psychological[All Fields] AND effects[All Fields])) AND (“mothers“[MeSH Terms] OR mother[Text Word])”. The polyglot search translator tool was used to adapt the search strategies across the databases [16]. The search included studies from inception until 2024. The search was last updated on 12th February 2025. The search was limited to the English language only.

### Selection of sources of evidence

The search results were imported to rayyan.ai [17] for deduplication. Two reviewers (GH & SSR) independently screened the titles and abstracts. Any discrepancy between the reviewers in the selection of articles was resolved by discussion with a third reviewer (KPK). The included list of articles was then taken up for full-text screening, which was independently reviewed by the two reviewers (GH & SSR), and if there were any disagreements, they were resolved by discussion with the third reviewer (KPK).

### Definition of psychological effects

In this review, psychological effects were defined as impacts on mothers’ mental state due to preterm birth. The psychological effects included symptoms of stress, anxiety, depression, postpartum depression, posttraumatic stress, and distress due to preterm birth.

### Charting the data

The data were extracted via a predesigned Microsoft Excel form, which included author, year of publication, country, study design, sample size, participant’s age, study duration, assessment time, psychological scales used, results, and risk factors. The data extraction was carried out by two authors (GH and SSR) independently, and if there were any conflicts, the data were resolved via the consensus-building approach.

### Collecting, summarizing, and reporting results

We used a narrative approach to summarize the findings, aided by tables and figures. The findings are presented with the study characteristics (study settings, design, sample size, and conclusions), scales used to measure the psychological effects (such as symptoms of distress, anxiety, depression, stress, postpartum depression, and posttraumatic stress) and risk factors predisposing individuals to the psychological effects.

## Results

### Study characteristics of the included studies

**Fig. 1:**
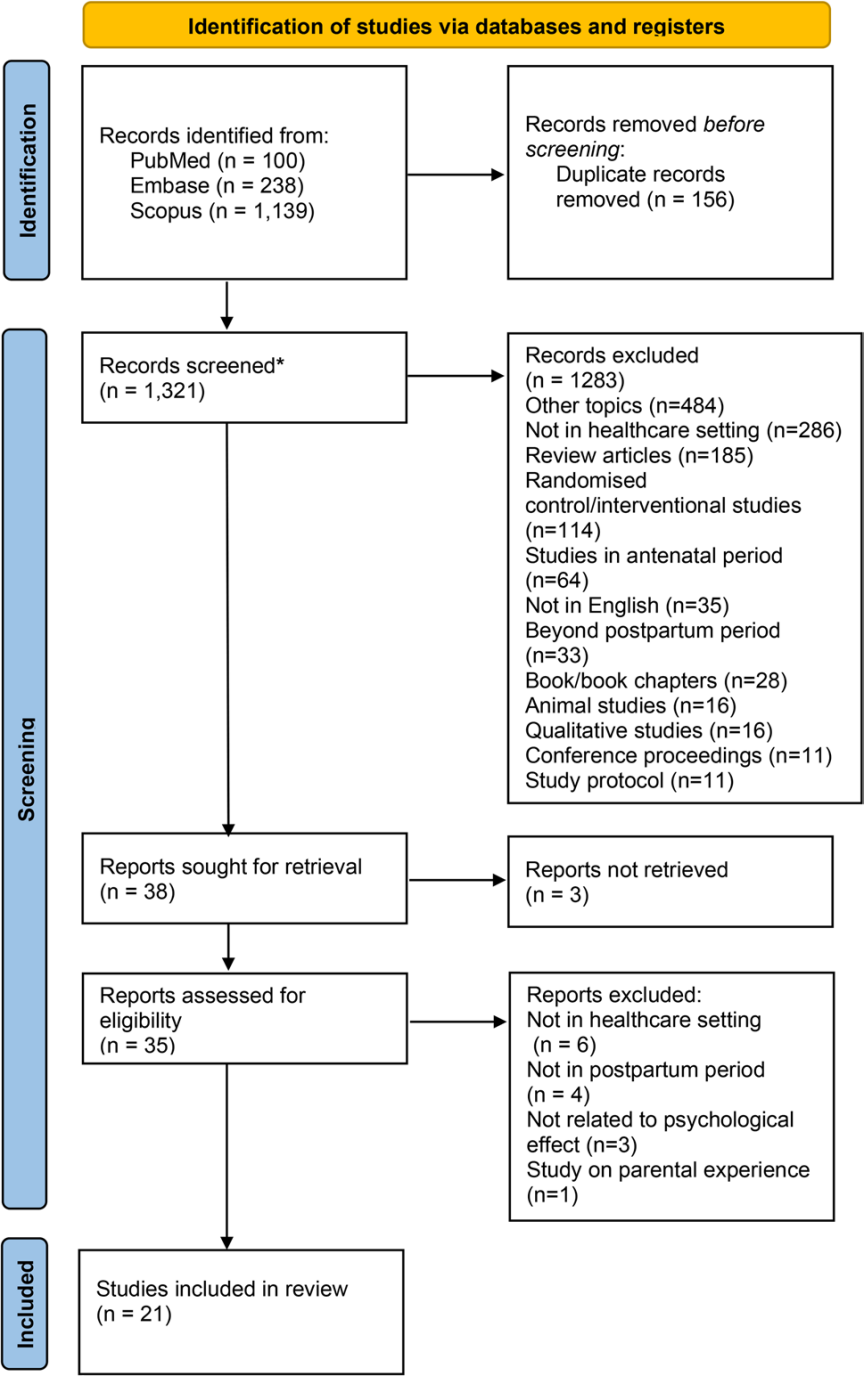
Prisma flow diagram depicting the screening process

Through electronic searches of PubMed (*n* = 100), Embase (*n* = 238), and Scopus (*n* = 1139), a total of 1477 articles were retrieved. Out of 1477 articles, 156 duplicates were removed via Rayyan. A total of 1321 articles were screened for title–abstract screening, and 35 articles were eligible for full-text screening. Out of 35 articles, 21 were included, and 14 were excluded because they were not in healthcare setting (*n* = 6), not in postpartum period (*n* = 4), not related to psychological effect (*n* = 3) or study on parental experience (*n* = 1). The PRISMA flow diagram depicting the screening process is presented in Fig. 1.

A total of 21 articles published across the globe were included. Five studies were conducted in Italy [18–22], and three studies were conducted in the United States [23–25]. Two studies were conducted in Australia [26, 27], and two studies were conducted in Norway [2, 28]. One study each was carried out in India [5], Canada [29], Greece [30], Qatar [31], Nigeria [32], Chile [6], France [33], Kenya [34], and Germany [35].

All the included studies were observational studies. A cross-sectional study design was most common (14 of 21, 66%), followed by a longitudinal study design (4 of 21, 19%), a cohort study design (2 of 21, 9%), and a case‒control study design (Table 1). Among the different scales used to screen for psychological effects, the state-trait anxiety inventory (STAI) was the most commonly used scale (8 of 21 studies), followed by the Center for Epidemiologic Studies Depression Scale (CES-D) (6 of 21 studies) (Supplementary file 2).

**Table 1:** Characteristics of the included studies

Study ID	Country	Study design	Sample size	Mothers age	Participants	Comparison	Study duration in months	Assessment time	Scales	Results	Predictors
Mira 2024 [6]	Chile	Cross-sectional	85	58.8% between 25–34	85 mothers of preterm infants	NA	10	2–3 weeks of hospitalization	PSS: NICU, EPDS	Parental role alterations were the most stressful area in PSS: NICU and 38.8% of mothers of preterm infants scored above cutoff in EPDS	Infant behavior and appearance
Deshwali 2023 [5]	India	Cross-sectional	260	28.1 ± 4.4	130 mothers of preterm infants	130 mothers of term infants	23	Within 1 week after delivery (preterm) and during discharge (term)	GAD-7, CES-D, SASRQ, PSS: NICU	GAD-7, CES-D scores were high in mothers with preterm neonates, and SASRQ was higher in mothers with term neonates. Sight and sound and parental role alterations were the most stressful area in PSS: NICU	Marital distress
McMahon 2023 [27]	Australia	Cohort study	143	32.8 ± 5.4	143 mothers of preterm infants	NA	25	Within 4 weeks from delivery (shortly after birth)	HADSA, CES-D	49% of mothers of preterm infants showed high levels of anxiety symptoms in HADSA and 41% of mothers of preterm infants had high levels of depressive symptoms in CES-D	NA
Blanc 2021 [33]	France	Cross-sectional	2270	29.9 ± 5.4	2270 mothers of preterm infants	NA	10	Within neonatal discharge	STAI, CES-D	83% of mothers of preterm infants showed STAI scores above cutoff score and 48.9% of mothers of preterm infants had scored above cutoff in CES-D	NA
Weigl 2020 [35]	Germany	Cross-sectional	46	30.5 ± 6.2	18 mothers of preterm infants	28 mothers of term infants	37	Within a week of delivery (six days)	STAI, BDI, PSS	BDI, STAI, and PSS scores were high in mothers with preterm neonates	NA
Mutua 2020 [34]	Kenya	Cross-sectional	172	41.3% between 20–25	86 mothers of preterm infants	86 mothers of term infants	NA	During hospitalization (preterm) and 6 weeks after delivery (term)	PHQ, EPDS, K10	Mothers of preterm infants had high levels of anxiety, postpartum depressive symptoms, and distress symptoms than in mothers of term infants	Gestational age, intimate partner violence
Pisoni 2019 [20]	Italy	Longitudinal	28	33.7 ± 4.50	20 mothers of preterm infants	20 mothers of term infants	6	During hospitalization	PSS: NICU, EPDS	Infant behavior and appearance and parenting role alterations were the most stressful area in PSS: NICU and postpartum depressive symptoms was high in mothers of preterm infants	Infant behavior and appearance
Duffy 2018 [25]	United States	Cross-sectional	52	27.7 ± 7.1	22 mothers of preterm infants	30 mothers of term infants	NA	At the time delivery	PSS	The scores were high in mothers of preterm infants	NA
Trumello 2018 [21]	Italy	Cross-sectional	62	33.98 ± 4.76	40 mothers of preterm infants delivered before 32 weeks	22 mothers of preterm infants delivered at or after 32 weeks	NA	Within 1 week after delivery	STAI, EPDS	Mothers of preterm infants delivered before 32 weeks had higher levels of anxiety symptoms and postpartum depressive symptoms than in mothers of preterm infants delivered at or after 32 weeks	Gestational age
Ionio 2017 [18]	Italy	Cross-sectional	81	35.29 ± 5.38	45 mothers of preterm infants	36 mothers of term infants	25	1–2 weeks from delivery	IES	Posttraumatic stress symptoms were not statistically significant between mothers of preterm and term infants	NA
Gondwe 2017 [23]	United States	Longitudinal	236	26.7 ± 6.3–28.7 ± 4.7	194 mothers of singletons preterm infants	42 mothers of multiple preterm infants	NA	At enrollment	CES-D, STAI, PPQ	CES-D, STAI and PPQ scores were higher in mothers with multiple preterm mothers, than in mothers with singleton preterm infants	NA
Ionio 2016 [19]	Italy	Cross-sectional	50	34.92 ± 4.56–36 ± 6.85	21 mothers of preterm infants	29 mothers of term infants	15	1–2 weeks from delivery	PSS: NICU, IES	Infants’ behavior and appearance and parental role alterations were the most stressful area in PSS: NICU and posttraumatic stress symptoms were not statistically significant between mothers of preterm and term infants	NA
Bouras 2015 [30]	Greece	Cross-sectional	200	32 ± 6.8	75 mothers of preterm infants	125 mothers of term infants	16	After 4 weeks of NICU admission, after a week (4 days) (term)	STAI, BDI	Mothers of preterm infants had higher anxiety and depressive symptoms above cutoff than in mothers of term infants	NA
Misund 2014 [2]	Norway	Cohort	29	33.7 ± 4.3	29 mothers of preterm infants	NA	38	When mothers were able to respond (4-30days)	STAI, IES, GHQ	17%, 65.5%, and 79.3% of mothers of preterm infants showed STAI, IES, and GHQ, respectively, scores above cutoff score,	Gestational age, previous childbirth
Misund 2013 [28]	Norway	Longitudinal	29	33.7 ± 4.3	29 mothers of preterm infants	NA	38	Within 2 weeks after delivery	STAI, IES, GHQ	STAI scores had significantly increased after discharge, IES and GHQ scores decreased after discharge	Preeclampsia, intraventricular hemorrhage, maternal age
Ballantyne 2013 [29]	Canada	Cross-sectional	291	31.64 ± 5.92–33.28 ± 5.76	107 immigrants’ mothers of preterm infants	184 Canadian mothers of preterm infants	21	During NICU discharge	CES-D, PSS: NICU	CES-D scores were high in immigrant mothers of preterm mothers than in Canadian mothers of preterm birth and PSS: NICU scores were quite similar in both these mothers	Single parent
Bener 2013 [31]	Qatar	Cross-sectional	1659	33.4 ± 6.1	170 mothers of preterm and low birth weight infants	1489 mothers of term infants	17	Unclear	DASS	Mothers of preterm infants had higher DASS scores above cutoff than in mothers of term infants	NA
Farnell 2012 [26]	Australia	Cross-sectional	52	17.12 ± 1.24–30.36 ± 6.01	13 adolescent mothers of preterm infants	13 adolescent mothers of term infants and 26 adult mothers of preterm infants	NA	Before a week of discharge or 2 days after delivery ( preterm) and 1 week after discharge ( term )	GHQ	GHQ scores were higher in adult mothers with preterm neonates than in adolescent mothers with preterm neonates and full-term neonates	NA
Gambina 2011 [22]	Italy	Case control study	84	32.9 ± 5.3	42 mothers of preterm infants	42 mothers of term infants	6	Within a week of delivery (three days) and before discharge	STAI, EPDS	STAI and EPDS scores above cutoff were higher in mothers of preterm infants than in mothers of term infants	NA
Ukpong 2011 [32]	Nigeria	Cross-sectional	57	28.6 ± 5.1	57 mothers of preterm infants	NA	5	During NICU discharge	HADS, GHQ	12.3%, 19.3%, and 36.8% of mothers of preterm infants had scored above cutoff in HADSA, HADSD, and GHQ, respectively	Gestational age, Birth weight
Mew 2003 [24]	United States	Longitudinal	39	29.0 ± 5.3	39 mothers of preterm infants	NA	NA	During enrollment	CES-D, PSS: NICU	50% of mothers of preterm infants had scored above cutoff in CES-D and infant behavior and appearance were the most stressful area	Infant behavior and appearance

*PSS NICU *Perceived Stress Scale Neonatal Intensive Care Unit, *EPDS *Edinburgh Postnatal Depression Scale, *GAD *Generalized Anxiety Disorder Scale, *CES-D* Center for Epidemiologic Studies-Depression Scale, *SASRQ* Standford Acute Stress Reaction Questionnaire, *HADSA* Hospital Anxiety and Depression Scale Anxiety, *STAI* State-Trait Anxiety Inventory, *BDI* Beck Depression Inventory, *PSS * Perceived Stress Scale, *PHQ* Patient Health Questionnaire, *K10 *Kessler Psychological Distress Scale, *IES* Impact of Event Scale, *PPQ *Perinatal Posttraumatic Stress Disorder Questionnaire, *GHQ *General Health Questionnaire, *DASS* Depression Anxiety Stress Scale, *HADS* Hospital Anxiety and Depression Scale

The psychological effects included anxiety, depressive symptoms, stress, posttraumatic stress symptoms, postpartum depressive symptoms, and distress symptoms. Thirteen of the 21 studies measured anxiety [2, 6, 21–23, 27, 28, 30–35]. Eleven studies screened for depressive symptoms [6, 23, 24, 27, 29–35]. Nine studies screened for stress [5, 6, 19, 20, 24, 25, 29, 31, 35]. Five studies each screened for posttraumatic stress symptoms [2, 18, 19, 23, 28], postpartum depressive symptoms [6, 20–22, 34], and distress symptoms [2, 26, 28, 32, 34].

### Mothers’ characteristics

A total of 5925 women participated in the included studies. Maternal age was reported in 19 of 21 studies, and the mean age ranged between 17.12 and 35.29 years.

### Measures of psychological effects

#### Anxiety

Among the 13 studies, six compared mothers with preterm and term infants. The percentage of anxiety was greater in mothers with preterm infants than in mothers with term infants, ranging from 17% to 75% [5, 22, 31, 34]. A study by Gondwe et al. compared mothers with singleton preterm infants with mothers with multiple preterm infants. Mothers with multiple preterm infants presented greater levels of anxiety symptoms than mothers with singleton preterm infants did [23]. A study by Trumello et al. compared mothers of preterm infants who delivered before 32 weeks with those who delivered at or after 32 weeks. The percentage of anxiety symptoms was greater in mothers of preterm infants who delivered before 32 weeks (72%) than in those who delivered at or after 32 weeks (42%) [21]. Five studies had no comparison group. Among these, the percentage of anxiety ranged from 12.3% to 83% in mothers with preterm infants [2, 27, 28, 32, 33].

#### Depression

Among the 11 studies, five compared mothers with preterm and term infants. The percentage of depressive symptoms was greater in mothers with preterm infants than in mothers with term infants, ranging from 29.4% to 90.6% [5, 30–32, 34]. In a study by Gondwe et al., mothers with singleton preterm infants were compared with mothers of multiple preterm infants, and mothers with multiple preterm infants presented greater levels of depressive symptoms than mothers with singleton preterm infants did [23]. A study by Ballantyne et al. compared preterm infants of immigrant mothers with preterm infants of Canadian-born mothers. The percentage of depressive symptoms was greater in immigrant mothers with preterm infants (59%) than in Canadian mothers with preterm infants (47%) [29]. Four studies had no comparison group. The percentage of mothers with preterm infants with depressive symptoms ranged from 19.3% to 50% [24, 27, 32, 33].

#### Stress

Among the nine studies, six compared mothers with preterm and term infants. Mothers of preterm infants had greater levels of stress than mothers of term infants did, ranging from 11.2% to 60% [19, 20, 25, 31, 35]. A study by Ballantyne et al. compared stress levels between preterm infants of immigrants and Canadian-born mothers, but the stress levels were quite similar in both groups [29]. Two studies had no comparison group; among them, Mira et al. reported that the percentage of stress was 92% in mothers of preterm infants [6, 24].

#### Posttraumatic stress

Among the five studies, two compared mothers with preterm and term infants. Mothers of preterm infants presented greater levels of posttraumatic stress symptoms than mothers of term infants did [18, 19]. A study by Gondwe et al. compared posttraumatic stress symptoms among mothers of singleton and multiple preterm infants, and the levels were greater in mothers of multiple preterm infants than in mothers of singleton preterm infants [23]. Two studies had no comparison group; among them, Misund et al. (2014) reported that the percentage of mothers with preterm infants with posttraumatic stress symptoms was 65.5% [2, 28].

#### Postpartum depression

Among the five studies, three compared mothers with preterm and term infants. The percentage of postpartum depressive symptoms was greater in mothers with preterm infants than in mothers with term infants, ranging from 19% to 85% [20, 22, 34]. A study by Trumello et al. compared mothers of preterm infants delivered before 32 weeks with those delivered at or after 32 weeks, and the percentage of postpartum depressive symptoms was greater in mothers of infants delivered before 32 weeks (68%) than in those delivered at or after 32 weeks (60%) [21]. A study by Mira et al. did not include a comparison group, and the percentage of mothers of preterm infants with postpartum depressive symptoms was 38.8% [6].

#### Distress

Among the five studies, one compared mothers with preterm and term infants. The percentage of distress symptoms was greater in mothers with preterm infants (75.6%) than in mothers with term infants (24.4%) [34]. A study by Farnell compared adolescent mothers of preterm infants with adolescent mothers of term infants and adult mothers of preterm infants, but the level of distress symptoms was high in adult mothers of preterm infants [26]. Three studies had no comparison group, and the percentage of distress ranged from 36.8% to 79.3% [2, 28, 32].

### Risk factors

**Fig. 2:**
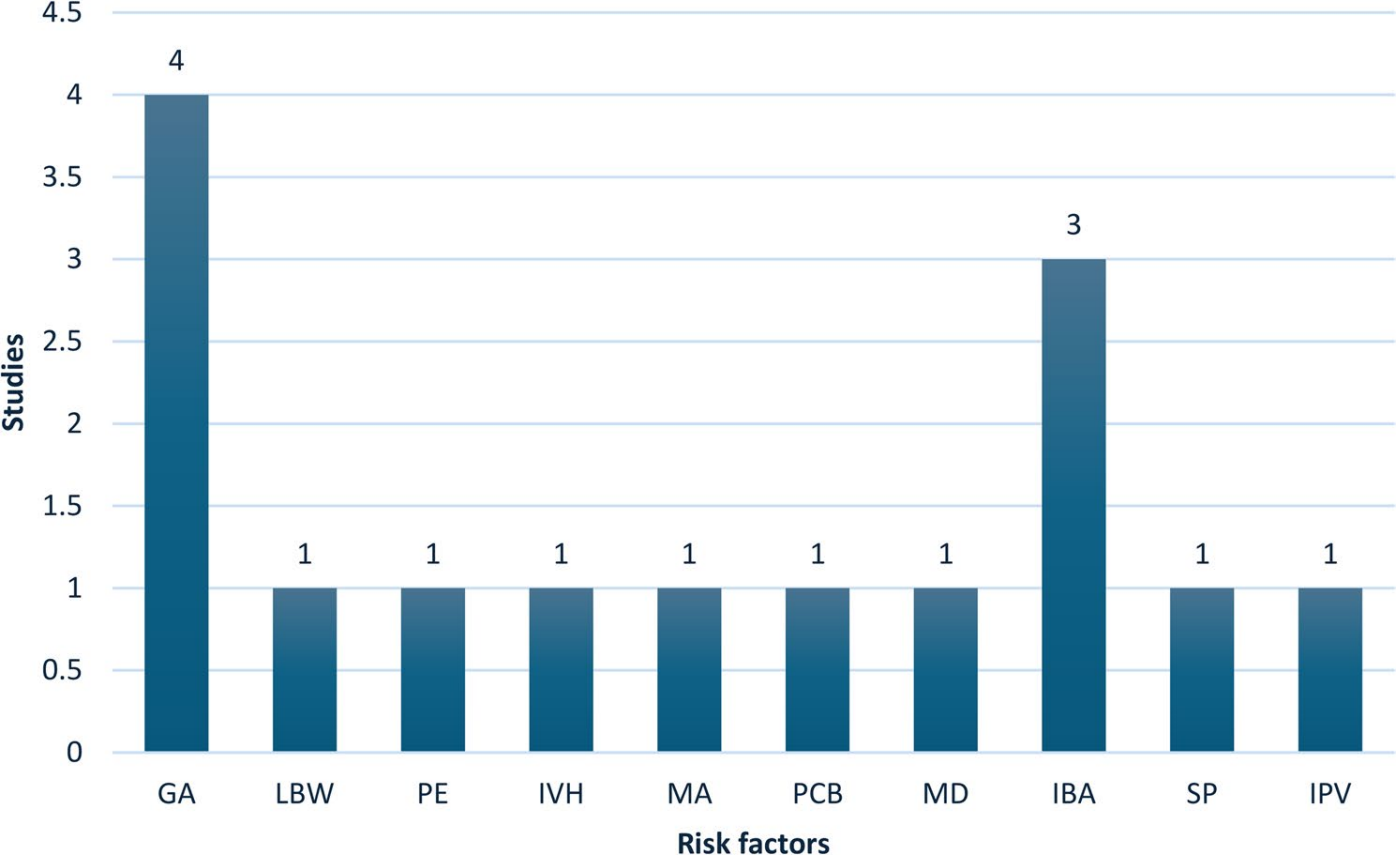
Risk factors for psychological conditions GA – Gestational age, LBW – Low birth weight, PE – Preeclampsia, IVH – Intraventricular hemorrhage, MA – Maternal age, PCB – Previous childbirth, MD – Marital distress, IBA – Infant behavior and appearance, SP – Single parent, IPV – Intimate partner violence

In this review several risk factors associated with adverse psychological outcomes in postnatal mothers following preterm birth were identified. Maternal factors such as increased maternal age, number of previous child births, preeclampsia, single parent, marital distress, and intimate partner violence contributed to increased psychological effects [2, 5, 28, 29, 34]. Gestational age was major risk factor, other neonatal factors were low birth weight, intraventricular hemorrhage (IVH), infant behavior and appearance in the NICU [2, 6, 20, 21, 24, 28, 32, 34]. Figure 2 depicts the risk factors.

## Discussion

This scoping review synthesized findings from 21 studies examining the psychological effects in mothers with preterm neonates within hospital settings and the tools utilized to screen these effects. Among the included studies, anxiety, depression and stress were the most frequently observed psychological effects, with a distinctly higher incidence among mothers with preterm infants than among mothers with term infants. These results highlight the considerable psychological burden linked with preterm birth and highlight the necessity for routine psychological screening during the hospitalization of these preterm infants.

The time-period dispersion of the included studies demonstrated how the problem changed over time. To be precise, six studies were conducted in the last 5 years, six in the last 10 years, eight studies were between 10 and 15 years, and only one study was conducted more than 20 years ago. This trend emphasizes the ongoing relevance of the problem across countries and the necessity of ongoing research, planning, and preventative measures.

Three of the 21 included studies were from low- and middle-income countries (LMICs). This is especially significant because preterm births are more prevalent in developing countries. The underrepresentation of LMICs in the literature draws attention to a significant research gap in the areas where the problem is most prevalent. This emphasizes the urgent need for increased investment in research, capacity development, and contextual studies in these nations to guide effective interventions and policies.

The discussion is organized around three main themes that were identified during the review to provide a thorough understanding of the findings.

### Theme 1: anxiety as the major psychological effect

Anxiety was the most often described psychological effect, identified in 13 studies, with a consistently higher prevalence among mothers of preterm infants than among mothers of term infants [5, 22, 31, 34]. Six studies comparing preterm and term infants reported anxiety levels ranging from 17% to 75% in mothers of preterm hospitalized infants compared with 4% to 25% in term infant mothers. This disparity indicated enhanced emotional vulnerability associated with preterm birth, exaggerated by uncertainty regarding infant outcomes and prolonged hospital stays. Anxiety symptoms frequently manifest intensely during the early postpartum period and during the NICU stay, when mothers feel uncertainty about their infant’s health, medical treatments, and survival.

The anxiety levels in LMICs ranged from 12.3% to 75% [5, 32, 34], whereas in developed countries, the levels ranged from 17% to 83%. This similar prevalence of anxiety in mothers of preterm infants indicates that anxiety is a global public health issue rather than one limited to particular economic or cultural settings.

Gondwe et al. reported higher levels of anxiety in mothers of multiple preterm infants than in those with singleton preterm infants, possibly due to greater caregiving duties and perceived risk [23]. Trumello et al. reported higher levels of anxiety in mothers who delivered before 32 weeks than in those who delivered later [21]. These findings imply that multiple births and gestational age are critical risk factors that influence anxiety in mothers with hospitalized preterm infants.

Anxiety is commonly assessed via the State Trait Anxiety Inventory (STAI), which measures both temporary state anxiety and stable trait anxiety.

These findings emphasize the need for routine psychological screening of mothers who are preterm in hospitalized settings. Collectively, these findings emphasize the necessity for routine psychological screening of mothers with preterm infants in hospital settings, especially during NICU stays. Tools such as the STAI must be integrated into clinical protocols. Tailored mental health services that consider gestational age and birth multiplicity are advocated.

Early detection of anxiety enables timely intervention, such as psychoeducation, counseling and relaxation-focused therapy. Research from intervention studies has demonstrated that increased parental involvement and activity-based group therapy can significantly reduce anxiety symptoms and promote coping in mothers [10].

### Theme 2: depressive symptoms and their persistence

Depressive symptoms or depression was the second most common mental health outcome, identified in 11 studies, and was most frequently measured by the CES-D and EPDS. The CES-D is a 20-item self-report questionnaire rated on a 4-point Likert scale that assesses depressive symptoms. Postnatal depression is commonly measured via the EPDS, a 10-item self-report questionnaire.

Ballantyne et al. reported greater depressive symptoms in immigrant mothers than in Canadian mothers [29], indicating the potential influence of cultural, social and systemic factors in mediating mental health outcomes. Gondwe et al. reported greater depressive symptoms in mothers with multiple preterm infants than in those with singleton preterm infants [23]. Longitudinal studies revealed that depressive symptoms may persist for months post-partum, highlighting the importance of continuing mental health care after hospital discharge [23, 24].

The depression levels in LMICs ranged from 19.3% to 77.6%, whereas in high-income countries, the levels ranged from 19% to 90.6%. The broad range in both settings suggests heterogeneity in maternal experiences and healthcare systems.

The high prevalence of depression in postnatal mothers following preterm birth highlights the need for screening for postpartum mothers via validated tools such as the CES-D and the EPDS. Socioeconomic and cultural differences in depressive symptoms indicate the need for support programs that are tailored specifically.

### Theme 3: other mental health outcomes

In the present review, maternal stress was reported in nine studies and was frequently assessed by the PSS: NICU or the Perceived Stress Scale. The PSS-NICU remains one of the tools most commonly used during the infant’s NICU stay, which is linked to changes in the behavior and appearance of the baby, the sights and sounds of the NICU and parental role alterations. The chief contributor to stress was the NICU environment, which is characterized by medical equipment, infant fragility, and limited parental involvement. Mira et al. reported that 92% of mothers with preterm infants reported high stress levels [6], which is quite alarming and advocates immediate strategies in health care settings. Ballantyne et al. reported that stress levels were similar in immigrant mothers and Canadian mothers, unlike depressive symptoms.

Symptoms of posttraumatic stress were identified in five studies and were commonly measured via the Impact of Event Scale (IES). Mothers with preterm infants had consistently greater levels of posttraumatic stress symptoms than did those with term infants. Studies by Ionio et al. and Misund et al. reported greater IES scores in mothers with preterm births [2, 18, 19]. Mothers with multiple preterm infants had more obvious PTSD symptoms, as reported by Gondwe et al., again highlighting the additional psychological burden of caring for more than one high-risk newborn [23].

Five studies reported on distress. Using the K10 scale, Mutua et al. reported that mothers of preterm infants had significantly greater levels of distress [34]. Misund et al. related distress to factors such as preeclampsia and invasive birth experiences [28].

The high levels of psychological distress, stress, and posttraumatic stress that have been observed in mothers with preterm infants highlight the urgent need for integrated mental health support in healthcare settings. Peer support groups, psychological counseling, and parental participation programs can help reduce emotional stress and enhance the health of mothers and their infants. Clinicians should modify care techniques to encourage family involvement, culturally sensitive communication, and emotional support, as the NICU setting itself is a significant stressor.

### Limitations

We used only three databases for searching, which is recognized as a limitation. Only articles in English were included; therefore, research that has been published in non-English literature is limited. In this review, 14 of the 21 included studies were cross-sectional, which limits causal interpretations and long-term outcomes. There was heterogeneity with respect to the psychological tools used and the timing of assessment, which limits the comparability of the results. Only quantitative studies were included. This review might have overlooked information from qualitative studies that provide readers with a different perspective on these issues that mothers face after preterm birth.

### Implications for clinical practice and future research

Healthcare providers, primarily staff and nurses in neonatal units, must prioritize psychiatric screening in mothers with preterm infants with validated tools such as the STAI, PSS: NICU, CES-D, and EPDS. These professionals should be trained to administer these screening tools effectively and analyze the results within the setting of early postpartum care. Policy makers should consider this screening as a part of routine screening of preterm mothers in national programs.

Future studies should explore longitudinal follow-ups for psychological screening beyond healthcare settings, that is, at home, as the chances of psychological reactions may be greater due to less support from medical professionals at home. Tailor made mental health support programs should be designed for at risk mothers. Ongoing regular maternal screening and support programs should be implemented along with preterm follow up.

## Conclusion

This review highlights that preterm birth has substantial psychological effects on mothers in healthcare settings, anxiety being the most commonly reported outcome, followed by depressive symptoms and stress. Most frequently used screening tools among the studies were the State-Trait Anxiety Inventory for anxiety, the Center for Epidemiology Studies Depression Scale for depression, and the Perceived Stress Scale: NICU for stress. Gestational age less than 37 weeks, increased maternal age, multiparity, preeclampsia, marital distress, single parent, intimate partner violence, low birth weight, intra ventricular hemorrhage, infant’s behavior and appearance in the NICU were identified as risk factors for psychological effects in postnatal mothers following preterm birth. Our findings emphasize on the need of psychological screening of postnatal mothers after preterm birth, addressing the at risk mothers with counselling and mental health support in order to improve maternal well-being and more favorable developmental trajectories for preterm infants.

## Supplementary Information

Supplementary Material 1.

Supplementary Material 2.

## Data Availability

The datasets used and/or analyzed during the current study are available from the corresponding author upon reasonable request.

## Abbreviations

NICU: Neonatal intensive care unit
JBI: Joanna Briggs Institute
PRISMA: Preferred reporting items for systematic reviews and meta–analyses
STAI: State Trait Anxiety Inventory
CES-D: Center for Epidemiologic Studies Depression Scale
PSS: NICU: Perceived stress scale: neonatal intensive care unit
HADSA: Hospital anxiety and depression scale–anxiety
GAD: Generalized anxiety disorder scale
EPDS: Edinburgh Postnatal Depression Scale
PHQ: Patient health questionnaire
K10: Kessler Psychological Distress Scale
PTSD: Posttraumatic stress disorder
IVH: Intraventricular hemorrhage
LMICs: Low–and Middle–Income Countries

## References

[1] Perinatal mental health [Internet]. [cited 2025 Jun 12]. Available from: https://www.who.int/teams/mental-health-and-substance-use/promotion-prevention/maternal-mental-health

[2] Misund AR, Nerdrum P, Diseth TH. Mental health in women experiencing preterm birth. BMC Pregnancy Childbirth. 2014;14:263. 25107462 10.1186/1471-2393-14-263 PMC4137092

[3] Yates R, Anderson PJ, Lee KJ, Doyle LW, Cheong JLY, Pace CC, et al. Maternal mental health disorders following very preterm birth at 5 years Post-Birth. J Pediatr Psychol. 2022;47(3):327–36. 34664642 10.1093/jpepsy/jsab101

[4] Ayele TB, Moyehodie YA. Prevalence of preterm birth and associated factors among mothers who gave birth in public hospitals of East Gojjam zone, Ethiopia. BMC Pregnancy Childbirth. 2023;23(1):204. 36964535 10.1186/s12884-023-05517-5 PMC10037778

[5] Deshwali A, Dadhwal V, Vanamail P, Sagar R, Sharma A, Agarwal R, et al. Prevalence of mental health problems in mothers of preterm infants admitted to NICU: A cross-sectional study. Int J Gynecol Obstet Off Organ Int Fed Gynecol Obstet. 2023;160(3):1012–9. 10.1002/ijgo.14466 36115010

[6] Mira A, Coo S, Bastías R. Mother’s mental health and the interaction with her moderate preterm baby in the NICU. J Reprod Infant Psychol. 2024;42(2):299–314. 35635499 10.1080/02646838.2022.2077921

[7] Hunt AM, Uthirasamy N, Porter S, Jimenez ME. Parental depression screening in pediatric health care settings: A scoping review. Pediatrics. 2022;150(1):e2021055804. 35762257 10.1542/peds.2021-055804

[8] White-Traut R, Norr KF, Fabiyi C, Rankin KM, Li Z, Liu L. Mother–infant interaction improves with a developmental intervention for mother–preterm infant dyads. Infant Behav Dev. 2013;36(4):694–706. 23962543 10.1016/j.infbeh.2013.07.004 PMC3858517

[9] Drury SS, Scaramella L, Zeanah CH. The Neurobiological impact of postpartum maternal depression: prevention and intervention approaches. Child Adolesc Psychiatr Clin N Am. 2016;25(2):179. 26980123 10.1016/j.chc.2015.11.001 PMC4794751

[10] John HB, Philip RM, Santhanam S, Padankatti SM, Sebastian T, Balan I, et al. Activity based group therapy reduces maternal anxiety in the neonatal intensive care Unit - a prospective cohort study. Early Hum Dev. 2018;123:17–21. 30031995 10.1016/j.earlhumdev.2018.07.001

[11] Martini J, Petzoldt J, Knappe S, Garthus-Niegel S, Asselmann E, Wittchen HU. Infant, maternal, and Familial predictors and correlates of regulatory problems in early infancy: the differential role of infant temperament and maternal anxiety and depression. Early Hum Dev. 2017;115:23–31. 28869923 10.1016/j.earlhumdev.2017.08.005

[12] Sandnes R, Le Floch M, Riquin E, Nocus I, Müller JB, Bacro F. Parental stress and mental health outcomes following very preterm birth: A systematic review of recent findings. J Affect Disord. 2024;355:513–25. 38556094 10.1016/j.jad.2024.03.154

[13] Peters M, Godfrey C, Mcinerney P, Munn Z, Trico A, Khalil H. Chapter 11: Scoping Reviews. In. 2020.

[14] Arksey H, O’Malley L. Scoping studies: toward a methodological framework. Int J Soc Res Methodol. 2005;8(1):19–32.

[15] Tricco AC, Lillie E, Zarin W, O’Brien KK, Colquhoun H, Levac D, et al. PRISMA extension for scoping reviews (PRISMA-ScR): checklist and explanation. Ann Intern Med. 2018;169(7):467–73. 30178033 10.7326/M18-0850

[16] Clark JM, Sanders S, Carter M, Honeyman D, Cleo G, Auld Y, et al. Improving the translation of search strategies using the polyglot search translator: a randomized controlled trial. J Med Libr Assoc JMLA. 2020;108(2):195–207. 32256231 10.5195/jmla.2020.834 PMC7069833

[17] Ouzzani M, Hammady H, Fedorowicz Z, Elmagarmid A. Rayyan—a web and mobile app for systematic reviews. Syst Rev [Internet]. 2016 [cited 2024 Aug 28];5(1). Available from: https://link.springer.com/epdf/10.1186/s13643-016-0384-4 10.1186/s13643-016-0384-4 PMC5139140 27919275

[18] Ionio C, Lista G, Mascheroni E, Olivari MG, Confalonieri E, Mastrangelo M, et al. Premature birth: complexities and difficulties in Building the mother-child relationship. J Reprod Infant Psychol. 2017;35(5):509–23. 29517381 10.1080/02646838.2017.1383977

[19] Ionio C, Colombo C, Brazzoduro V, Mascheroni E, Confalonieri E, Castoldi F, et al. Mothers and fathers in NICU: the impact of preterm birth on parental distress. Eur J Psychol. 2016;12(4):604–21. 27872669 10.5964/ejop.v12i4.1093 PMC5114875

[20] Pisoni C, Spairani S, Manzoni F, Ariaudo G, Naboni C, Moncecchi M, et al. Depressive symptoms and maternal psychological distress during early infancy: A pilot study in preterm as compared with term mother-infant dyads. J Affect Disord. 2019;257:470–6. 31310909 10.1016/j.jad.2019.07.039

[21] Trumello C, Candelori C, Cofini M, Cimino S, Cerniglia L, Paciello M, et al. Mothers’ depression, anxiety, and mental representations after preterm birth: A study during the infant’s hospitalization in a neonatal intensive care unit. Front Public Health. 2018;6(DEC):1–9. 30581812 10.3389/fpubh.2018.00359 PMC6293875

[22] Gambina I, Soldera G, Benevento B, Trivellato P, Visentin S, Cavallin F, et al. Postpartum psychosocial distress and late preterm delivery. J Reprod Infant Psychol. 2011;29(5):472–9.

[23] Gondwe KW, Yang Q, White-Traut R, Holditch-Davis D. Maternal psychological distress and Mother-Infant relationship: Multiple-Birth versus Singleton preterm infants. Neonatal Netw NN. 2017;36(2):77–88. 28320494 10.1891/0730-0832.36.2.77

[24] Morawski Mew A, Holditch-Davis D, Belyea M, Shandor Miles M, Fishel A. Correlates of depressive symptoms in mothers of preterm infants. Neonatal Netw. 2003;22(5):51–60. 14598980 10.1891/0730-0832.22.5.51

[25] Duffy AR, Schminkey DL, Groer MW, Shelton M, Dutra S. Comparison of hair cortisol levels and perceived stress in mothers who deliver at preterm and term. Biol Res Nurs. 2018;20(3):292–9. 29490472 10.1177/1099800418758952

[26] Farnell L, Jones L, Rowe J, Sheeran N. Effects of age and the Pre-Term birth of an infant on adolescent mothers’ psychological adjustment. Child Health Care. 2012;41(4):302–21.

[27] McMahon GE, Treyvaud K, Spittle AJ, Giallo R, Lee KJ, Cheong JL, et al. Parental mental health and parenting behaviors following very preterm birth: associations in mothers and fathers and implications for child cognitive outcome. J Pediatr Psychol. 2023;48(3):293–304. 36655518 10.1093/jpepsy/jsac094

[28] Misund AR, Nerdrum P, Bråten S, Pripp AH, Diseth TH. Long-term risk of mental health problems in women experiencing preterm birth: a longitudinal study of 29 mothers. Ann Gen Psychiatry. 2013;12(1):33. 24176131 10.1186/1744-859X-12-33 PMC4175092

[29] Ballantyne M, Benzies KM, Trute B. Depressive symptoms among immigrant and Canadian born mothers of preterm infants at neonatal intensive care discharge: a cross sectional study. BMC Pregnancy Childbirth [Internet]. 2013;13 Suppl 1. Available from: https://www.scopus.com/inward/record.uri?eid=2-s2.0-84881359703&doi=10.1186%2f1471-2393-13-s1-s11&partnerID=40&md5=3b5a6e39c05e4ad24cf5e62f9b812ef6 10.1186/1471-2393-13-S1-S11 PMC3561187 23445606

[30] Bouras G, Theofanopoulou N, Mexi-Bourna P, Poulios A, Michopoulos I, Tassiopoulou I, et al. Preterm birth and maternal psychological health. J Health Psychol. 2015;20(11):1388–96. 24323334 10.1177/1359105313512353

[31] Bener A. Psychological distress among postpartum mothers of preterm infants and associated factors: a neglected public health problem. Rev Bras Psiquiatr Sao Paulo Braz 1999. 2013;35(3):231–6. 10.1590/1516-4446-2012-0821 24142082

[32] Ukpong DI. Factors associated with psychological morbidity in mothers of preterm infants: A study from Wesley guild Hospital, Nigeria. J Obstet Gynecol. 2011;31(2):146–8. 21281031 10.3109/01443615.2010.538773

[33] Blanc J, Rességuier N, Lorthe E, Goffinet F, Sentilhes L, Auquier P, et al. Association between extremely preterm Cesarean delivery and maternal depressive and anxious symptoms: a National population-based cohort study. BJOG Int J Obstet Gynecol. 2021;128(3):594–602. 10.1111/1471-0528.16499 32931138

[34] Mutua J, Kigamwa P, Ng’ang’a P, Tele A, Kumar M. A comparative study of postpartum anxiety and depression in mothers with preterm births in Kenya. J Affect Disord Rep. 2020;2:100043.

[35] Weigl T, Schneider N, Stein A, Felderhoff-Müser U, Schedlowski M, Engler H. Postpartal affective and endocrine differences between parents of preterm and Full-Term infants. Front Psychiatry. 2020;11:251. 32296356 10.3389/fpsyt.2020.00251 PMC7139630

