# Supplementary material for: The psychological effects of preterm birth on postnatal mothers: a scoping review
Source: BMC Pregnancy Childbirth. 2025 Nov 22;26:25. doi: 10.1186/s12884-025-08504-0 (PMC12777424; doi:10.1186/s12884-025-08504-0)
Supplement: Supplementary file 2 — Supplementary Material 2. [file 12884_2025_8504_MOESM2_ESM.docx]

Table 2: Measures of psychological effects

| Psychological effect | Scale | Version | Cutoff | Mean scores | Observations | Studies |
| --- | --- | --- | --- | --- | --- | --- |
| Anxiety | State-trait anxiety inventory (STAI) | STAI 40 | 45 | 41.56±13.41 | Mothers of preterm infants higher percentage of anxiety compared to mothers of term infants | (Bouras et al., 2015) |
|  |  | STAI 40 Italian validated version | 45 | 49.5 ± 9 | Mothers of preterm infants (17%) had higher percentage of anxiety above cutoff compared to mothers of term infants (4%) | (Gambina et al., 2011) |
|  |  | STAI 40 Italian validated version | 39/40 | 48.55±13.68 | Mothers of preterm infants delivered before 32 weeks (72%) had higher rates of anxiety symptoms compared to those delivered at or after 32 weeks (45%) | (Trumello et al., 2018) |
|  |  | STAI 40 French validated version | 45 | NA | 83% of mothers of preterm infants showed STAI scores above cutoff score | (Blanc et al., 2021) |
|  |  | STAI-S 20 | 40 | Singleton 40.43 ± 13.67  Multiple 43.66 ± 11.13 | Mothers of multiple preterm infants had significantly higher anxiety symptoms than mothers of singleton infants | (Gondwe et al., 2017) |
|  |  | STAI-S 20 | 40 | NA | Mothers of preterm infants had significantly higher anxiety than term mothers | (Weigl et al., 2020) |
|  |  | STAI 10 (own version) | 20 | 21.5±2.8 | 66% had psychological effects at 2 weeks postpartum whereas it was 31% at 18 moths | (Misund et al., 2013) |
|  |  | STAI 10 (own version) | 20 | 21.45±0.53 | 17% of mothers of preterm infants showed STAI scores above cutoff score | (Misund et al., 2014) |
|  | Hospital Anxiety and Depression Scale | HADS-A 7 | 8 | 7.69±3.96 | 49% of mothers of preterm infants showed high levels of anxiety symptoms | (McMahon et al., 2023) |
|  |  | HADSA 7 | 8 | 3.8±2.3 | 12.3% of mother of preterm infants showed high levels of anxiety | (Ukpong, 2011) |
|  | Depression Anxiety Stress Scale | DASS 7 | 8 | NA | Mothers of preterm infants (26.5%) had higher percentage of anxiety above cutoff compared to mothers of term infants (11.6%) | (Bener, 2013) |
|  | Generalized Anxiety Disorder Scale | GADS 7 | 10 | 10.6 ± 3.5 | Mothers of preterm infants (66.2%) had higher percentage of anxiety symptoms above cutoff compared to mothers of term infants (46.9%) | (Deshwali et al., 2023) |
|  | Patient Health Questionnaire 4 | PHQ 2 (A) | 3 | NA | Mothers of preterm infants (75%) had higher percentage of anxiety compared to mothers of term infants (25%) | (Mutua et al., 2020) |
| Depression | Center for Epidemiologic Studies-Depression scale | CES-D 20 | 16 | 20.73 ± 11.41 | Immigrant mothers of preterm infants (59%) had higher percentage of depressive symptoms above cutoff compared to Canadian mothers of preterm infants (47%) | (Ballantyne et al., 2013) |
|  |  | CES-D 20 | 16 | 18.0 ± 4.8 | Mothers of preterm infants (54.6%) had higher percentage of depressive symptoms above cutoff compared to mothers of term infants (23.1%) | (Deshwali et al., 2023) |
|  |  | CES-D 20 | 16 | Singleton 16.47±11.73  Multiple 17.58±11.92 | Mothers of multiple preterm infants had significantly higher depressive symptoms than mothers of singleton infants | (Gondwe et al., 2017) |
|  |  | CES-D 20 | 16 | 17.9 ± 10.1 | 50% of mothers of preterm infants had scored above cutoff | (Morawski Mew et al., 2003) |
|  |  | CES-D 20 | 16 |  | 48.9% of mothers of preterm infants had scored above cutoff score | (Blanc et al., 2021) |
|  |  | CES-D 16 | 16 | 15.34±9.98 | 41% of mothers of preterm infants had high levels of depressive symptoms | (McMahon et al., 2023) |
|  | Beck Depression Inventory | BDI 21 | 18 | 10.26 ±7.88 | Mothers of preterm infants (90.6%) had higher percentage of depressive symptoms above cutoff compared to mothers of term infants (85.6%) | (Bouras et al., 2015) |
|  |  | BDI 21 | >11 | NA | 55% of mothers of preterm infants scored above cutoff | (Weigl et al., 2020) |
|  | Hospital Anxiety and Depression Scale | HADSD | 8 | 3.9±3.2 | 19.3% of mothers of preterm infants had high levels of depression | (Ukpong, 2011) |
|  | Patient Health Questionnaire 4 | PHQ 2 | 3 | NA | Mothers of preterm infants (75%) had higher percentage of depression compared to mothers of term infants (25%) | (Mutua et al., 2020) |
|  | Depression Anxiety Stress Scale | DASS 7 | 10 | NA | Mothers of preterm infants (29.4%) had higher percentage of depression above cutoff compared to mothers of term infants (17.3%) | (Bener, 2013) |
| Stress | Parental Stress Scale: Neonatal Intensive Care Unit | PSS: NICU 34 | 35 | 3.22±0.81 | The scores were quite similar for both immigrant and Canadian born mothers | (Ballantyne et al., 2013) |
|  |  | PSS: NICU 34 | 35 | Total 9.27±2.24  PRA 3.06±0.86  IBA 3.73±0.94 | Infant behavior and appearance were the most stressful area | (Ionio et al., 2016) |
|  |  | PSS: NICU 34 Chile version | NA | PRA 3.89±0.81  SS 2.83±1.04 | 92% of mothers of preterm infants reported stress | (Mira et al., 2024) |
|  |  | PSS: NICU 28 | NA | PRA 31±4.7  IBA 45.3±8.7  SS 18.5±4.6 | Infant behavior and appearance were the most stressful area | (Morawski Mew et al., 2003) |
|  |  | PSS: NICU 26 | NA | SS 4.3±0.3  IBA 3.8±0.4  PRA 4.3±0.5 | Parental role alterations and sight and sound were the most stressful area | (Deshwali et al., 2023) |
|  |  | PSS: NICU 26 Italian version | ≥3 per scale | Total 3.11±0.98  SS 2.46±1.01  IBA 3.17±1.05  PRA 3.42±1.04 | 60% of mothers of preterm infants had scored above the cutoff score | (Pisoni et al., 2019) |
|  | Perceived Stress Scale | PSS 20 | NA | NA | The scores were significantly higher in the mothers of preterm infants compared to mothers of term infants | (Weigl et al., 2020) |
|  |  | PSS 14 | 19 | 25.91 ± 7.0 | The scores were significantly high in mothers of preterm infants compared to mothers of term infants | (Duffy et al., 2018) |
|  | Standford Acute Stress Reaction Questionnaire | SASRQ 30 | ≥3 | NA | Mothers of term infants (6.2%) had higher percentage of stress symptoms above cutoff compared to mothers of preterm infants (5.4%) | (Deshwali et al., 2023) |
|  | Depression Anxiety Stress Scale | DASS 7 | 15 | NA | Mothers of preterm infants (11.2%) had higher percentage of stress above cutoff compared to mothers of term infants (8.5%) | (Bener, 2013) |
| Posttraumatic stress | Impact of Event Scale | IES 22 | NA | Total 2.79±1.89  Intrusion 1.32±0.62  Hyperarousal 0.90±0.79 | There was no significant difference between mothers of preterm and term infants | (Ionio et al., 2016) |
|  |  | IES 22 | NA | Avoidance 0.61±0.53  Intrusion 1.13±0.63  Hyperarousal 0.62±0.28 | There was no significant difference between mothers of preterm and term infants | (Ionio et al., 2017) |
|  |  | IES 15 | 8 per scale | Total 19.7±10.8 | 66% had psychological effects at 2 weeks postpartum whereas it was 31% at 18 moths | (Misund et al., 2013) |
|  |  | IES 15 | 8 per scale | Total 19.66±2  Intrusion 14±1.48  Avoidance 5.66±0.81 | 65.5% of mothers of preterm infants had symptoms of posttraumatic stress | (Misund et al., 2014) |
|  | Perinatal Posttraumatic Stress Disorder Questionnaire | PPQ 14 | NA | Singleton 4.44±3.29  Multiple 5.68±2.23 | Mothers of multiple preterm infants had significantly higher posttraumatic stress symptoms than mothers of singleton infants | (Gondwe et al., 2017) |
| Postpartum depression | Edinburgh Postnatal Depression Scale | EPDS 10 Italian version | 10 | 9.5±4.5 | Mothers of preterm infants (19%) had higher percentage of postpartum depression above cutoff compared to mothers of term infants (2%) | (Gambina et al., 2011) |
|  |  | EPDS 10 | 13 | 11.5±5.95 | Mothers of preterm infants (85%) had higher percentage of postpartum depressive symptoms compared to mothers of term infants (25%) | (Pisoni et al., 2019) |
|  |  | EPDS 10 | 8/9 | 13.05±5.25 | Mothers of preterm infants delivered before 32 weeks (68%) had higher rates of postpartum depressive symptoms compared to those delivered at or after 32 weeks (60%) | (Trumello et al., 2018) |
|  |  | EPDS 10 Chile | 13 | NA | 38.8% of mothers of preterm infants scored above cutoff | (Mira et al., 2024) |
|  |  | EPDS 10 | 13 | NA | Mothers of preterm infants (77.6%) had higher percentage of depressive symptoms compared to mothers of term infants (22.4%) | (Mutua et al., 2020) |
| Distress | General Health Questionnaire | GHQ 30 | 6 | 40.10±2.90 | 79.3% of mothers of preterm infants had symptoms of distress | (Misund et al., 2014) |
|  |  | GHQ 30 | 6 | 40±15.4 | 66% had psychological effects at 2 weeks postpartum whereas it was 31% at 18 moths | (Misund et al., 2013) |
|  |  | GHQ 30 | 5 | 4.2±4.9 | 36.8% of mothers of preterm infants had high levels of distress | (Ukpong, 2011) |
|  |  | GHQ 12 | NA | 13.23±4.91 | Symptoms of distress were more in adult mothers with preterm infants compared to adolescent mothers with preterm infants and adolescent mothers with term infants | (Farnell et al., 2012) |
|  | Kessler psychological distress scale | K10 | 20 | NA | Mothers of preterm infants (75.6%) had higher percentage of distress symptoms compared to mothers of term infants (24.4%) | (Mutua et al., 2020) |
